# A New Method of Inducing Anæsthesia Preparatory to Operations on the Teeth, in the Gazette de Hopitaux

**Published:** 1859-04

**Authors:** 


*A new Method of inducing Anæsthesia preparatory to Operations on the Teeth, in the Gazette de Hopitaux.*—M. Claisee, reports a new method of inducing local anæsthesia. Having introduced into a small bottle camphor enough to fill one-third its capacity, he fills it full of sulphuric ether, and allows it to remain till the camphor is dissolved. He then dips into this solution, a sponge attached to a delicate piece of whalebone, rubs the gum with it, preparatory to the incision. The anæsthesia passes off in ten minutes.

